# Novel Polymeric Biomaterial Based on Naringenin

**DOI:** 10.3390/ma14092142

**Published:** 2021-04-23

**Authors:** Malgorzata Latos-Brozio, Anna Masek, Małgorzata Piotrowska

**Affiliations:** 1 Faculty of Chemistry, Institute of Polymer and Dye Technology, Lodz University of Technology, Stefanowskiego 12/16, 90-924 Lodz, Poland; anna.masek@p.lodz.pl; 2 Faculty of Biotechnology and Food Sciences, Institute of Fermentation Technology and Microbiology, Lodz University of Technology, Wólczańska 71/173, 90-924 Lodz, Poland; malgorzata.piotrowska@p.lodz.pl

**Keywords:** naringenin, natural polyphenol, polymerization, cross-linking compound

## Abstract

Biomaterials prepared based on raw plant materials are becoming more and more popular due to their specific properties and environmental friendliness. Naringenin is a flavonoid naturally occurring in citrus fruit with antioxidant and pharmacological activity. Polymeric materials based on flavonoids may have favorable properties in comparison to monomeric polyphenols, such as stronger antioxidant and antimicrobial properties. One of the methods of obtaining the polymeric form of flavonoids is polymerization with a cross-linking compound. This method has already been used to obtain poly(quercetin) and poly(rutin) from a flavonol group as well as poly(catechin) from the flavan-3-ol group of flavonoids. However, to date, no polymeric forms of flavanones have been prepared in a cross-linking reaction; the aim of this study was to obtain poly(naringenin) by reaction with a cross-linking compound using glycerol diglycide ether GDE. The degree of conversion of naringenin to poly(naringenin) determined by FTIR spectroscopy was 85%. In addition, the thermal, antioxidant and antimicrobial properties of poly(naringenin) were analyzed. Poly(naringenin) was characterized by greater resistance to oxidation and better thermal stability than monomeric naringenin. Moreover, polymeric naringenin also had a better ability to scavenge ABTS and DPPH free-radicals. In contrast to monomeric form, poly(naringenin) had antimicrobial activity against *Candida albicans*. Polymeric biomaterial based on naringenin could potentially be used as a natural stabilizer and antimicrobial additive for polymer compositions, as well as pro-ecological materials.

## 1. Introduction

Naringenin (4,5,7-trihydroxy-flavanone) is one of the naturally occurring flavonoids from the polyphenol group. This flavanone is found mainly in citrus fruits, such as grapefruit (43.5 mg/100 mL), orange (2.13 mg/100 mL of juice) and lemon (0.38 mg/100 mL of juice) [1,2,3,4]. Naringenin can exist in two forms of aglycone or its glycosidic form (naringenin-7-O-glucoside). The formation of naringenin and other flavonoids in plants and fruits depends on many factors, including plant genetics and environmental conditions (soil type and light), as well as the degree of ripeness, processing and storage [5]. The pharmacological activity of naringenin has been proven. This compound has antioxidant, anti-inflammatory, neuroprotective, anti-cancer, anti-atherosclerotic and anti-diabetic properties [6,7,8,9,10,11].

**Figure 1:**
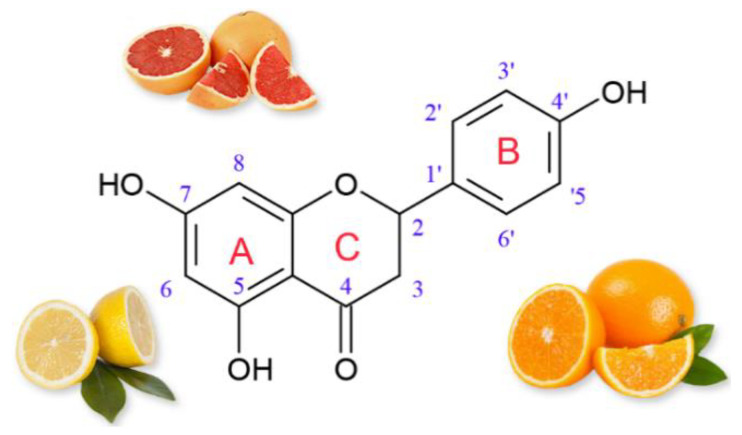
Structural formula and natural sources of naringenin.

The properties of naringenin, including its pharmacological activity, depend on the arrangement of correlated functional groups in its structure. Hydroxyl (OH) groups are characterized by significant reactivity against reactive oxygen species (ROS) as well as reactive nitrogen species. The 5,7-m-dihydroxy arrangement in ring A (Figure 1) is used to stabilize the structure of this flavonoid after donating electrons to free radicals. The association between 5-OH and 4-oxo substituents provides naringenin with the ability to chelate heavy metals [12,13].

The unique properties of flavonoids, including naringenin, are closely connected to their chemical structure [14,15]. Literature data indicate that the polymeric forms of flavonoids can be described by stronger antiradical properties, higher antimicrobial activity and better thermal stability [16,17]. Several reactions of flavonoid polymerization are known: polymerization using enzymes [18,19,20,21], photopolymerization [22], HCl acid catalyzed polymerization [23], self-condensation [24,25] and polymerization with a crosslinker [26,27]. Nevertheless, the polymerization of naringenin has not been described.

The aim of this study is polymerization of naringenin with a crosslinker (glycerol diglycide ether GDE), as well as analysis of the polymeric form of flavonoids in terms of thermal properties, antioxidant capacity and antimicrobial activity.

Glycerol diglycidyl ether (GDE) is a polyfunctional cross-linking compound. According to the literature data [27], this compound is a biocompatible agent and can be used to convert monomeric forms of flavonoids into polymeric structures. The phenolic OH groups in the flavonoids readily react with the epoxy groups in GDE to form polymeric structures [26,27].

The method of polymerizing flavonoids with a cross-linking agent, GDE, was only used to obtain polymeric forms of quercetin [26]; its glycoside, rutin [27]; and also (+)-catechin [28]. However, a method for obtaining the polymeric forms of naringenin by reaction glycerol diglycidyl ether (GDE) as a crosslinking agent has not as yet been described.

## 2. Materials and Methods

### 2.1. Preparation of Polymeric Form of Naringenin in Reaction with Cross-Linking Agent

A naringenin (natural, 98%, MW: 272.25 g/mol, Sigma Aldrich, Shanghai, China,) reaction with glycerol diglycidyl ether (GDE) crosslinker was performed according to the method described by Sahiner [26,27] with minor modifications.

In the first step, a solution of naringenin was prepared by dissolving 1 g in 10 mL of 1 M NaOH (ChemPur, Piekary Śląskie, Poland). Dissolving the naringenin in NaOH was one of the elements of the reaction, which was aimed at increasing the solubility of this compound, as well as opening the rings before polymerization (cross-linking reaction). In the next step, 4 mL of naringenin solution was added to 150 mL of a 0.1 M solution of L-α-lecithin (from soybean, ≥99%, MilliporeSigma, Darmstadt, Germany) in cyclohexane (96%, pure. P.A., ChemPur, Poland). Then, the mixture was stirred for 2 h at 1000 rpm at room temperature. After that time, glycerol diglycidyl ether (technical grade, Sigma-Aldrich, Darmstadt, Germany) was added in an amount of 100 mol% with respect to naringenin used. The solution was stirred for 2 h at 1000 rpm and 25 °C. Poly(naringenin) was washed twice with cyclohexane by centrifugation (6000 rpm, 20 °C) and dried at 35 °C for 72 h in a dryer.

### 2.2. Infrared FTIR and UV-Vis Spectroscopy of Naringenin and Poly(naringenin)

FTIR analysis: A Nicolet 670 FTIR spectrophotometer (Thermo Fisher Scientific, Waltham, MA, USA) was used to examine the structure of poly(flavonoid). Powders of monomeric naringenin and poly(naringenin) were placed at the output of infrared beams. The examination of oscillating spectra allows definition of the functional groups with which the radiation interacted. Based on the FTIR spectra, the degree of naringenin to poly(naringenin) conversion was determined.

UV-Vis analysis: The spectra of naringenin and poly(naringenin) samples at wavelengths of 190 to 1100 nm were recorded utilizing a UV-Vis spectrophotometer (Evolution 220, Thermo Fisher Scientific, Waltham, MA, USA).

### 2.3. Microscopic Analysis of Naringenin and Poly(naringenin)

Digital microscopy: photos of naringenin and poly(naringenin) samples were obtained using a digital microscope VHX-7000 at magnifications of 20 to 400 times.

Scanning electron microscopy (SEM): based on the photos received from a scanning electron microscope (SEM) LEO 1530 (Carl Zeiss AG, Oberkochen, Germany), the morphology of naringenin and poly(naringenin) powders was assessed. Magnification was 1000, 10,000 and 25,000×.

### 2.4. Thermal Analysis (TG and DSC) of Naringenin and Poly(naringenin)

Thermogravimetric analysis (TG): the thermal stability of naringenin and poly(naringenin) was measured using a Mettler Toledo Thermobalance (TA Instruments, Greifensee, Switzerland). Powders of flavonoid and poly (flavonoid) (10 mg) were placed in alumina crucibles and heated from 25 °C to 800 °C under argon flow (50 mL/min) at a rate of 5 °C/min.

Differential scanning calorimetry (DSC): temperature ranges of naringenin and poly(naringenin) phase changes were carried out utilizing a Mettler Toledo DSC analyser (TA 2920; TA Instruments, Greifensee, Switzerland). Powders weighing 5 to 6 mg were placed in 100 μL aluminum pans and heated from −80 to 400 °C at a rate of 10 °C/min in air.

### 2.5. Antioxidant Capacity

The ability of naringenin and poly(naringenin) to reduce free radicals was determined using ABTS and DPPH tests. The activity of these compounds in reducing transition metals was tested by FRAP (for iron ions) and CUPRAC (for cupric ions) methods.

The authors presented a detailed description of these analytical tests in a previous publication that also concerned polymerization with a crosslinker [28].

ABTS and DPPH methods: tests were based on the reduction of radicals ABTS (2,2′-azino-bis(3-ethylbenzothiazoline-6-sulphonic acid)) and DPPH (2,2-diphenyl-1-picrylhydrazyl). The level of inhibition (%) of free radicals ABTS and DPPH was computed according to the following Equation (1):

Inhibition of free radicals (%) = [((A_0_ − A_1_)/A_0_) × 100] (1)

where A_0_ is the absorbance of the reference sample without naringenin and poly(naringenin), and A_1_ is the absorbance in the presence of naringenin or the polymeric form of naringenin [28]. Absorbance was measured at 734 nm for the ABTS method and at 517 nm for the DPPH test using a UV spectrophotometer (Evolution 220, Thermo Fisher Scientific, Waltham, MA, USA).

The inhibition level of free radicals (%) was computed utilizing a standard curve prepared with Trolox. The effect of naringenin and poly(naringenin) on the reduction of ABTS and DPPH radicals is referred to as the Trolox equivalent antioxidant capacity (TEAC).

FRAP and CUPRAC tests assay: The FRAP (ferric reducing antioxidant power) test is based on the reduction of the ferric ion (Fe^3+^→Fe^2+^) under acidic conditions. The CUPRAC (cupric reducing antioxidant capacity) test is similar to the FRAP method and consists of the reduction of Cu^2+^ to Cu^+^. The ferric (FRAP) and cupric (CUPRAC) ions’ reducing capacity was computed according to Equation (2):

ΔA = A_AR_ − A_0_ (2)

where: A_0_ is the absorbance of the reagent sample, A_AR_ is the absorbance after reaction [28]. The absorbance was measured at 595 nm for the FRAP assay and at 450 nm for the CUPRAC determination using a UV spectrophotometer.

Naringenin and poly(naringenin) solutions with a concentration of 1.0 mg/mL were prepared for the ABTS, DPPH, FRAP and CUPRAC tests. Naringenin molecules are insoluble in water but soluble in organic solvents such as alcohol [29], so it was dissolved in ethanol for this work. Poly(naringenin) was insoluble in ethanol, but partially soluble in distilled water; therefore, the poly(flavonoid) solution was prepared with water.

### 2.6. Antimicrobial Properties

Antimicrobial tests were performed in accordance with ASTM E2149: Standard Test Method for Determining the Antimicrobial Activity of Antimicrobial Agents Under Dynamic Contact Conditions, American Society for Testing and Materials (ASTM International) [30]. Antibacterial and anti-fungal tests were determined utilizing the dynamic “flask shake method”. Bacterial test strains such as *Escherichia coli* ATCC 8739, *Staphylococcus aureus* ATCC 6538 and *Bacillus subtilis* ATCC 6633, as well as fungi *Candida albicans* ATCC 10231 and *Aspergillus niger* ATCC 16404 were used for antimicrobial tests. The cultures were stored on slants with Merck’s TSA (bacteria) and MEA (fungi) medium at 6 °C. Before the experiment, the strains were activated. Samples of naringenin and poly(naringenin) (10 mg) were placed in test tubes. The 9.9 mL of nutrient broth, and then 0.1 mL of a suspension of test microorganisms suspended in physiological saline were added to the test tubes.

In the next step, prepared test tubes were incubated under dynamic conditions on a shaker (150 rpm) for 24 h at 30 °C (*B. subtilis* and *A. niger*) and 37 °C (other strains).

The number of microorganisms in the samples after 24 h of incubation was determined by the culture test on TSA (bacteria) and MEA (fungi). Furthermore, the control samples (only microorganisms) were counted at the start of the experiment (t = 0). The results are assumed as the number of colony-forming units/mL of the medium (cfu/mL). The dieback rates of microorganisms D were calculated from Equation (3):

D = (log number of microorganisms _t = 0_ − log number of microorganisms _t = 24_) (3)

### 2.7. Statistical Analysis

Determinations of antioxidant activity and antimicrobial tests were carried out on three control samples, and the average results were reported. Computations were carried out for the means and standard deviations of three independent tests (*n* = 3). Statistical analysis was used for comparison of the means using a Fischer LSD test (the significance level was set at *p* < 0.05).

## 3. Results and Discussion

**Figure 2:**
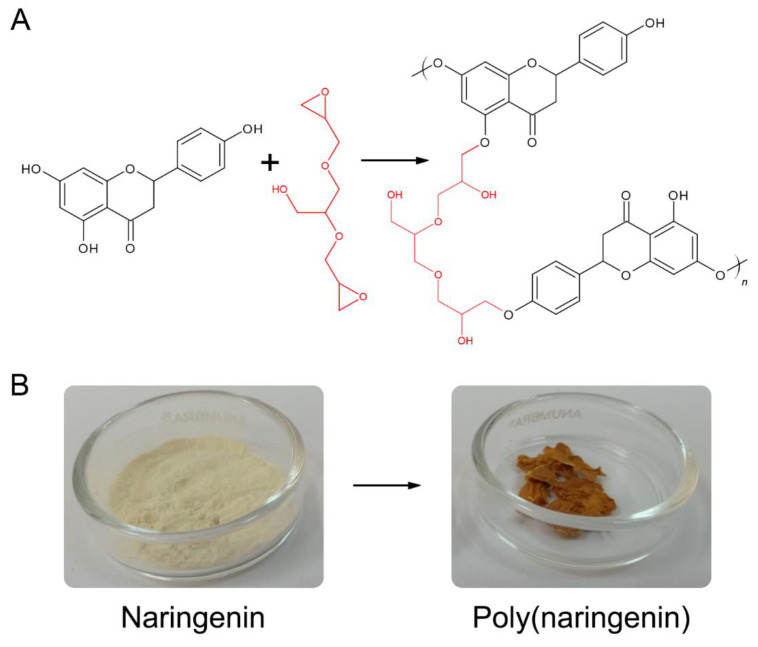
Proposed scheme of cross-linking reaction of naringenin with glycerol diglycidyl ether (GDE), where n—number of mers in poly(naringenin) (**A**); visual change of color of naringenin during polymerization with GDE (digital camera photos, Canon INC, Tokio, Japan) (**B**).

Figure 2a suggests the cross-linking reaction of naringenin with glycerol diglycidyl ether (GDE). The aliphatic epoxy monomer GDE can be used as a diepoxy cross-linking agent. GDE has good adhesion as well as good thermomechanical properties. This cross-linking compound is proposed for use in the synthesis of epoxy materials that can be applied in biodegradable materials [31,32]. The phenolic OH groups in naringenin can readily react with the epoxy groups in the GDE cross-linking agent. As a result of the reaction, polymer molecules of naringenin were formed.

There is little literature data on the cross-linking of flavonoids and its mechanism [26,27,28]. Articles on cross-linking of querectin [26], rutin [27] and catechin [28] have been published. Due to the lack of precise literature data, it is difficult to say whether the crosslinking is specific for OH groups in positions 5 and 4′ of quercetin or whether it can be in other OH groups, as well as whether the crosslinking of rutin is specific for positions 7 and 3′. The cited information is consistent with the cross-linking mechanisms proposed by Sahiner and co-authors.

However, the specificity of crosslinking of flavonoids may depend on the electron density and the presence of groups having an electron donation effect. Based on quantum-chemical calculations [33], the HOMO and LUMO energies were calculated. The HOMO and LUMO energies indicate the weakest and most susceptible places to oxidation and other reactions in the analyzed chemical compound. The computed energy of the highest filled orbital (E_HOMO_—ionization potential) determines the characteristic tendency of the molecule to donate electrons. For quercetin, it was found that OH groups in positions 5 and 4′ have an electron-donating effect, which may determine crosslinking in these positions.

In the case of rutin polymerization, determining the specificity of crosslinking is more difficult due to the presence of glycoside, which is steric hindrance. It was observed that the major role in rutin anti-radical activities was played by free hydroxyl groups on C4′, C3′ and C7 [21]. However, little data are available on the behavior of these activities during polymerization. It seems that these reactive groups may also play an important role in the mechanism of cross-linking rutin.

Besides poly(naringenin), the authors also investigated poly(catechin) [28]. Catechin and naringenin belong to different groups of flavonoids. Naringenin described in the manuscript is a flavanone. Catechin belongs to the flavan-3-ol group. Unlike catechin, the structure of naringenin is based on a carbon skeleton with a ketone group in position 4, in ring C. Moreover, in ring B, catechin has two OH groups in the 4′ and 5′ position, and naringenin has only one OH group in the 4′ position.

Due to the differences in the structure of flavonoids, the cross-linking reaction may proceed according to different patterns. In the article [28], the catechin cross-linking reaction involving OH groups in the 5, 4′ and 5′ position was proposed. However, in this manuscript, a reaction scheme with the participation of OH groups in the 5, 7 and 4′ position was suggested for naringenin. Different propositions of the catechin and naringenin reactions result from the structural differences of both compounds and may be related to the electron density of the compounds.

Quantum chemical calculations [34] showed that the OH groups in ring B are most easily susceptible to oxidation in the catechin molecule. The E_HOMO_ for catechin was −8.646 eV. The computed energy of the highest filled orbital (E_HOMO_—ionization potential) determined the ease of electron release and indicates the sites most susceptible to oxidation.

Due to the quoted quantum chemical calculations for catechin, it seems that the proposed scheme of compound cross-linking with the participation of OH groups from the B-ring is justified. The scheme of the naringenin crosslinking reaction contained in the manuscript is suggested and one of many possible reactions. Quantum chemical calculations and electrochemical studies can be helpful in predicting the mechanisms of naringenin polymerization.

**Figure 3:**
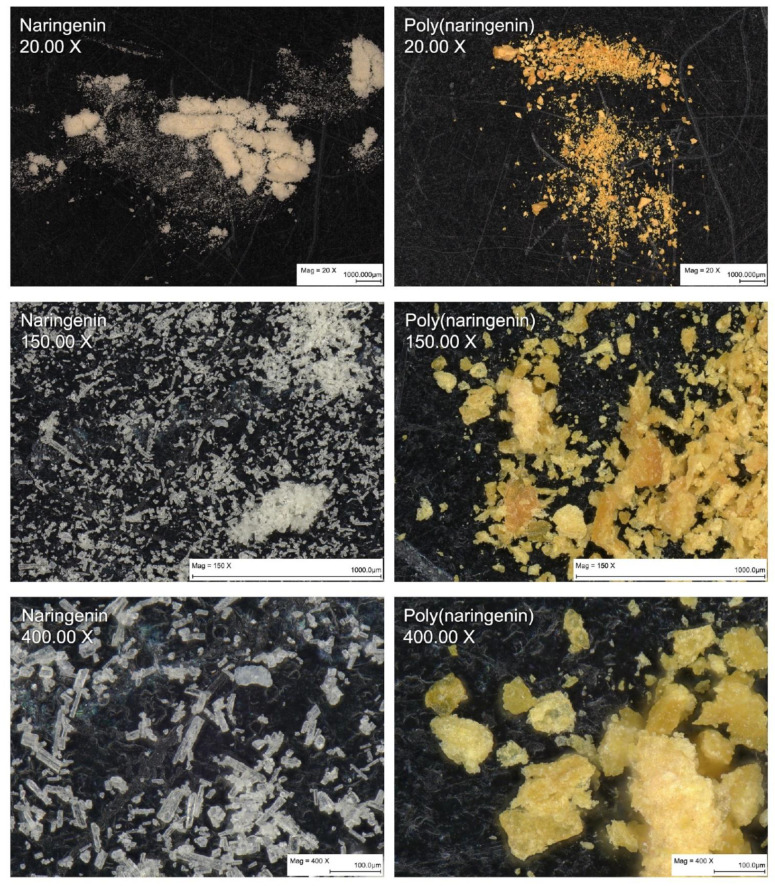
Digital microscope images of naringenin and polymeric naringenin at 20×, 150× and 400× magnification.

**Figure 4:**
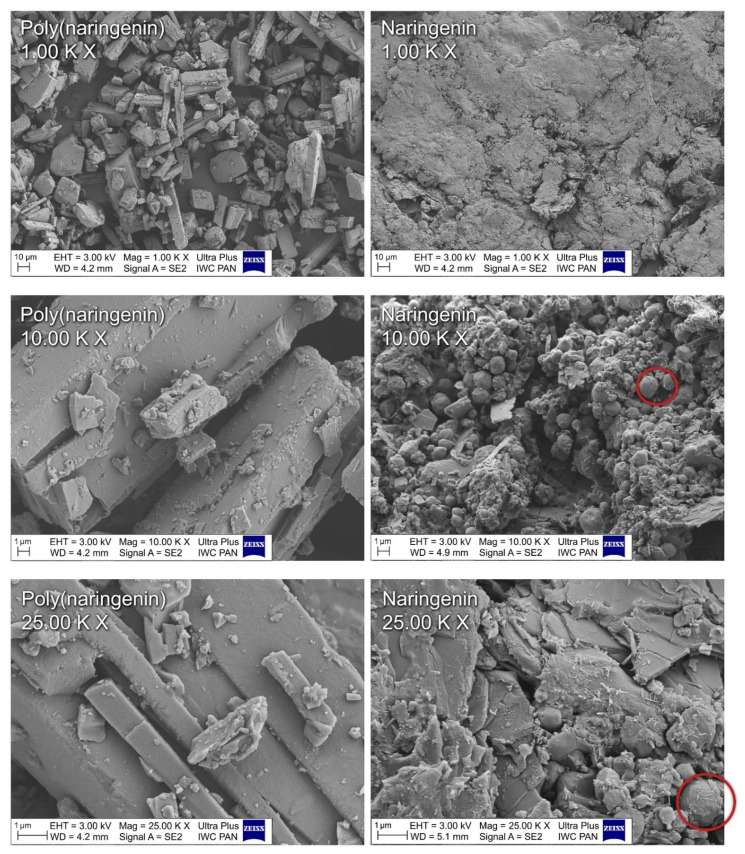
SEM images of naringenin and poly(naringenin) at 1.00 K×, 10.00 K× and 25.00 K× magnification. Red circles: ball-shaped structures.

During the poly(flavonoid) formation reaction, a clear change in color of naringenin was observed (Figure 2b), as well as a change in its powder morphology (Figure 3 and Figure 4). Monomeric naringenin was characterized by a milky-white color, while poly(naringenin) was yellow-orange (Figure 2b and Figure 3).

On the basis of photos taken with an optical microscope (Figure 3) and SEM (Figure 4), changes in naringenin morphology after polymerization were examined. Monomeric naringenin was characterized by a needle-like structure. The polymeric flavonoid was more compact and lamellar (Figure 3 and Figure 4). During the reaction with GDE, the naringenin molecules were linked together by epoxy groups. In the SEM pictures of poly(naringenin) (Figure 4), in contrast to the monomeric needle-shaped flavonoid, platelets and ball-shaped structures covered with thin needles were visible. Ball-shaped structures (marked with red circles on Figure 4) could be characterized for the GDE agent. Similar observations were found for polymeric forms of (+)-catechin obtained by cross-linking with GDE [28].

In order to analyse the structure of poly(naringenin), samples for liquid NMR were prepared. Unfortunately, the polymeric naringenin powder was only partly soluble in deuterated water, DMF and DMSO, which made liquid NMR tests impossible. The ^1^H NMR spectra of poly(naringenin) in deuterated water and DMSO are publicised in the Supplementary Materials (Figure S1a,b). The signals corresponding to the deuterated water (at 4.8 ppm) and DMSO (at 2.5 ppm; 3.3 ppm and 4.8 ppm) were obtained in the ^1^H NMR spectra.

Moreover, the ^1^H NMR spectra showed weak peaks in the range of 6.5 to 7.5 ppm corresponding to aromatic compounds. Signals at about 5 ppm can be related to OH groups. These peaks may correspond to unreacted naringenin monomers that dissolved in the test solutions.

Signals in the range of 2.3 to 3.8 ppm may correspond to functional groups in the GDE cross-linking compound, which, similar to monomeric naringenin, may not completely reactivate. Compared to the very pronounced solvent peaks, the mentioned peaks were very faint and were interpreted as impurities or residual naringenin monomer and GDE.

A high degree of cross-linking of poly(naringenin) may cause the limited solubility of the compound; generally, cross-linked polymers are not soluble. However, the limited solubility significantly hinders the analysis of the structure of poly(naringenin). A similar limited solubility of polymeric flavonoid was found for poly(catechin) received in the cross-linking reaction with GDE agent. In the ^1^H NMR spectra of the poly(catechin) obtained in the cross-linking reaction, only peaks corresponding to the solvents deuterated water and DMSO were visible [28].

**Figure 5:**
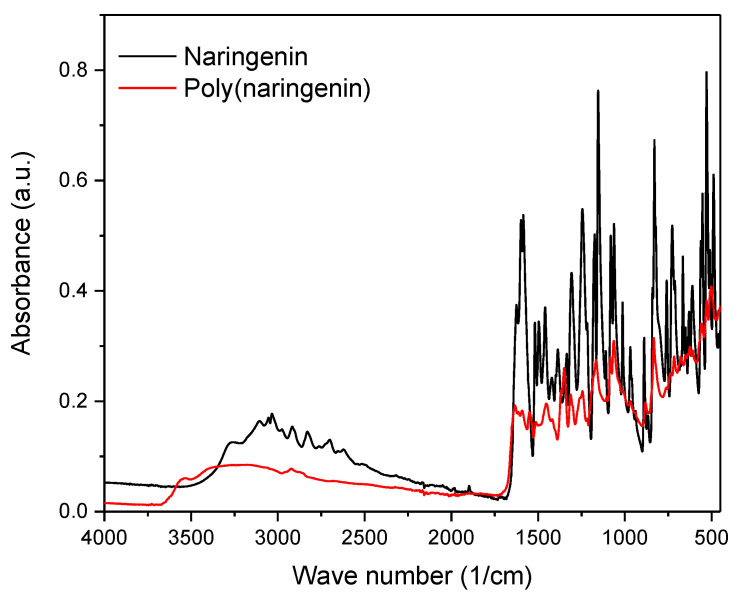
FTIR spectra of naringenin and poly(naringenin).

The polymeric form of poly(naringenin) powder was confirmed by FTIR spectroscopy (Figure 5). According to the literature, the polymerized flavonoids have characteristic functional groups present in the FTIR spectrum [26,27,28]. As with polymeric quercetin [26], rutin [27] and catechin [28], poly(naringenin) had specific bands indicating the macromolecular form of the compound: 3700 to 3000 cm^−1^-wide bands specific to the formation of free OH derived from glycerol diglycidyl ether; 1370 to 1250 cm^−1^ aryl stretching vibrations; 1061 cm^−1^ corresponding to C-CO-C in ketones; 750 to 790 cm^−1^ and 800 to 900 cm^−1^ corresponding to epoxies from glycerol diglycidyl ether. Moreover, peaks specific to the functional groups in monomeric flavonoids were visible on the FTIR spectrum [35]: 2922 cm^−1^ Ar-CH_3_; 1560 to 1570 cm^−1^ and 1450 to 1500 cm^−1^ (aromatic ring vibration) as well as 1165 cm^−1^ corresponding to C–OH. The cross-linking reaction of naringenin was confirmed by the appearance of bands corresponding to the functional group of polymeric flavonoids in the FTIR spectrum (Figure 5).

The conversion of naringenin to poly(naringenin) was calculated from the FTIR spectra. The peak at 1455 cm^−1^, corresponding to the vibration of the aromatic ring, did not change after the cross-linking reaction with GDE and was, thus, used as an internal reference [36,37]. The appearance of novel aryl bonds is specific for the polymerization of naringenin as well as other poly (flavonoids) such as quercetin, rutin and catechin [26,27,28]. Therefore, the peak at 1307 cm^−1^ was used to compute the conversion rate. During the calculations, the heights of the peaks were measured in centimeters from the baseline to the maximum point of the absorbance band. The degree of conversion of naringenin to poly(naringenin) was calculated using the following Equation (4):

(4) $$DC\;\left(\%\right)=\left(\frac{\frac{h_{1307}}{h_{1455}}\;poly\left(naringenin\right)}{\frac{h_{1307}}{h_{1455}}\;naringenin}\right)\times 100\%$$

where $h_{1307}$ is the height of the band at 1307 cm^−1^, and $h_{1455}$ is the height of the band at 1455 cm^−1^.

The degree of conversion of naringenin to poly(naringenin) was 85%. This high conversion degree can indicate good polymerization efficiency of naringenin with the GDE crosslinker compound. For comparison, the degree of conversion was 90% during the polymerization of catechin with GDE [28].

**Figure 6:**
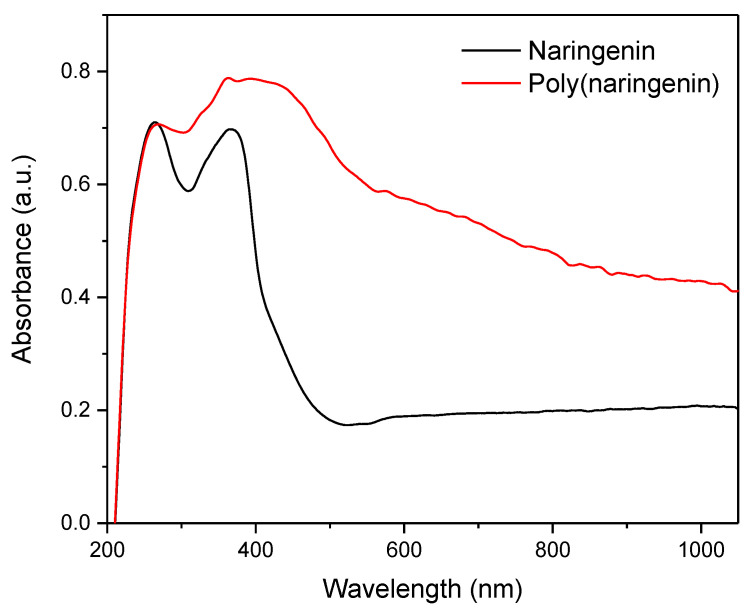
UV-Vis spectra of naringenin and poly(naringenin) powders. In the next stage of research, the thermal properties of naringenin and poly(naringenin) were investigated. The thermal stability of monomeric and polymeric forms of the flavonoid were established using thermogravimetry, TG. The results of the examinations are shown in Figure 7 and Table 1.

The polymeric naringenin was also confirmed by UV-Vis spectroscopy (Figure 6). The naringenin spectrum had two specific peaks at 200 to 300 nm and 300 to 400 nm. The poly(naringenin) powder was also characterized by two peaks: the peak between 200 and 300 nm, and a broad band between 300 and 550 nm. Literature reports suggest that the broad peak between 300 and 550 nm corresponds to the polymeric form of flavonoids, i.e., oligomeric catechin (300 to 550 nm) [38] and poly(catechin) received in the photopolymerization (300 to 750 nm) [16]. Similar results were obtained also for the poly(catechnin) obtained with the cross-linking compound. The polymeric form of catechin was characterized by two peaks, the first with a maximum at 250 nm and the second broad peak in terms of 300 to 900 nm [28].

Monomeric naringenin decomposed in one step, with a 67% weight loss of the sample in the temperature range of 290 to 380 °C. In contrast, the poly(naringenin) sample decomposed in two steps. The first step of decomposition occurred at around 250 °C, and the weight loss was 10%. The second step of decomposition occurred in the temperature range of 270 to 370 °C and was accompanied by a weight loss of the sample of 36.2%.

Table 1 shows the temperatures at which the weight loss of the samples amounted to 10% (T10), 50% (T50) and 60% (T60). Decomposition of poly(naringenin) started at a lower temperature than that of naringenin (T10 poly(naringenin) = 144 °C, T10 naringenin = 307 °C). The temperature for 50% decomposition of poly(naringenin), T50, was 20 °C lower compared to the T50 of monomeric naringenin. The final decomposition temperature, T60, of the polymeric naringenin was 94 °C higher than the T60 of naringenin, which meant higher thermal stability of the polymeric naringenin form. The increased thermal stability of poly(naringenin) may be the result of the cross-linked structure of the flavonoid, which reduces heat ingress into the molecules. In addition, in the polymeric form of naringenin, there can be fewer unbound functional groups that are less thermally resistant than those connected by nodes, which may also improve the thermal stability of the poly(naringenin).

**Figure 8:**
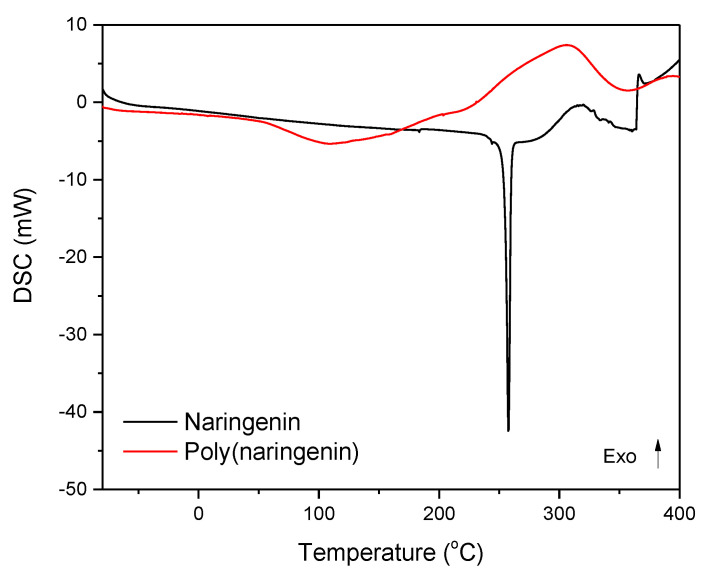
DSC thermograms of naringenin and poly(naringenin).

The poly(naringenin) powder obtained was exposed to differential scanning calorimetry (DSC). The powders were heated from −80 to 400 °C at a rate of 10 °C min in an air atmosphere. For comparison, the DSC of the reference naringenin was performed. The results are shown in Figure 8 and Table 2.

The monomeric naringenin thermogram shows an endothermic peak specific to melting of the sample, and an exothermic peak related to flavonoid oxidation. The thermogram of poly (naringenin) also showed an endothermic peak corresponding to the sample melting, and an exothermic peak of poly (flavonoid) oxidation.

Poly(naringenin) had a lower melting point than the monomeric form of flavonoid (T_m_ naringenin = 253.9 °C; T_m_ poly(naringenin) = 45.1 °C). This was related to the addition of the GDE cross-linking agent which can lower T_m_. The enthalpy of melting of poly(naringenin) (425.1 J/g) was about 2.6 times higher than the enthalpy of melting of naringenin (163.2 J/g). Poly(naringenin) had a higher final oxidation temperature T_o_ (by 13.8 °C) and a higher enthalpy of oxidation ΔH_o_ (approximately 6.8 times) than monomeric flavonoid. Thus, the polymeric form of naringenin showed better resistance to oxidation. The improved resistance to oxidation of the poly (flavonoid) may be due to the lower amount of unbound functional groups that react with oxygen. Functional groups susceptible to oxidation are strongly connected by network nodes, so their oxidation is limited and difficult.

The analyses of TG and DSC showed the higher thermal stability of poly (naringenin) as well as the increased resistance of the polymeric flavonoid to oxidation. The GDE used in the cross-linking reaction allows a poly (flavonoid) to be obtained, characterized by a lower initial decomposition temperature T10 (TG) and a lower melting point T_m_ (DSC) than naringenin. Similar results have been reported for the poly(catechin) obtained during cross-linking with GDE. Most importantly, both poly(catechin) [28] and poly(naringenin) had a higher final decomposition temperature and a higher oxidation temperature than the monomeric forms of flavonoids. The cross-linked structure of flavonoids undoubtedly improves their thermal properties by limiting the access of heat and oxygen to poly(flavonoid) particles and slowing down the processes of thermal decomposition and oxidation.

**Figure 9:**
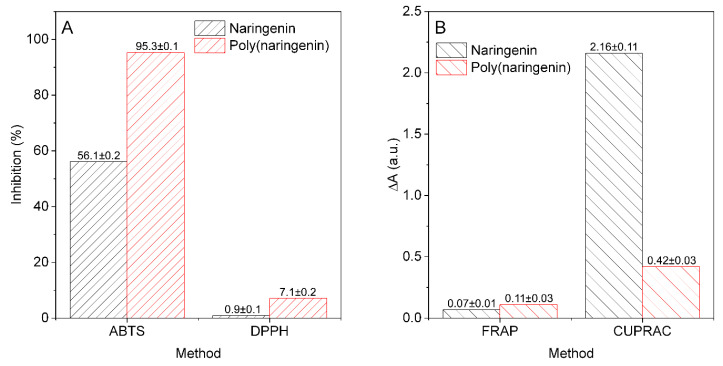
Ability of 1 mg/mL naringenin and poly(naringenin) to reduce radicals ABTS and DPPH (**A**) and for reduction of Fe^3+^ and Cu^2+^ ions measured by FRAP and CUPRAC tests (**B**).

Figure 9 shows the antioxidant activity of naringenin and poly(naringenin). Naringenin solutions in ethanol and poly(flavonoid) solutions in distilled water were prepared for ABTS, DPPH, FRAP and CUPRAC analyses. Different solvents were used due to the limited solubility of the test compounds, which is explained in detail in the methodology (Section 2.5). The concentrations of the solutions were 1 mg/mL. Both solvents—water and ethanol—are standard solvents used in the analysis of the antioxidant activity of natural compounds.

Apart from various solvents, the results of the antioxidant activity tests could have been influenced by the limited solubility of the compounds tested. Naringenin had good solubility in ethanol, while poly(naringenin) only was only partially soluble in H_2_O. Moreover, the poly(naringenin) solution had an intense yellow color, which could also adversely affect the results of spectrophotometric colorimetric tests.

Additionally, electrochemical tests were attempted for poly(naringenin); however, due to the insolubility in the standard acetonitrile solvent, the determination was not possible.

It is difficult to correctly assess the antioxidant activity of poly(naringenin) due to the incomplete solubility in solvents commonly used for the analysis of antiradical properties and the reduction of metal ions.

The ABTS method is dedicated to the analysis of hydrophobic and hydrophilic antioxidants. However, the analogous DPPH method is only used for the analysis of hydrophobic compounds. Naringenin had a good ability to reduce ABTS radicals (56.1 ± 0.2%; TEAC 29.3 ± 0.1 mmolT/100 g) and a negligible ability to reduce DPPH (0.9 ± 0.1%; TEAC 0.8 ± 0.1 mmolT/100 g) radicals. The polymeric form of naringenin, obtained as a result of cross-linking with GDE, showed greater activity towards scavenging both ABTS and DPPH radicals (ABTS: 95.3 ± 0.1%, TEAC 103.1 ± 1.0 mmolT/100 g; DPPH: 7.1 ± 0.2%, TEAC 26.9 ± 0.9 mmolT/100 g). The available literature data concern only the relationship between the structure and activity of monomeric naringenin (SAR) [9,12,13]. There is a lack of data on SAR relationships in polymeric flavonoids. Moreover, determination of such relationships is difficult and may be biased with some error due to the poor solubility of poly(naringenin). Limited solubility significantly hindered the determination of antioxidant properties with spectrophotometric methods. The activity of naringenin in relation to reactive oxygen species and reactive nitrogen species is related to the presence of hydroxyl substituents (OH) in its molecule [9]. The introduction of additional OH groups to the poly(naringenin), as a result of the polymerization reaction, could contribute to the improvement of antioxidant properties measured by ABTS and DPPH methods. Monomeric naringenin may have a smaller number of reactive OH groups in total than the polymerized form. The cross-linking reaction of naringenin with the participation of OH groups in the 5, 7, 4′ position is a proposed polymerization with GDE (Figure 2a). It cannot be ruled out that the cross-linking reaction will proceed according to a different pattern; therefore, other structural elements may also be responsible for the antioxidant activity. In the naringenin molecule, the 5,7-m-dihydroxy arrangement in ring A serves to stabilize the structure after donating electrons to free radicals. Polymeric naringenin may contain more 5,7-m-dihydroxy arrangements in rings A than the monomeric form and, therefore, may have a greater ability to reduce ABTS and DPPH radicals.

Comparatively, analysis of the antioxidant activity of poly(catechin) obtained with GDE showed that the polymeric flavonoid had improved activity for reducing ABTS free radicals and worse for reducing DPPH. It was found that the results of the ABTS and DPPH methods can indicate a greater affinity of polymeric catechin to the ABTS test, designed for the analysis of hydrophilic and hydrophobic compounds, unlike the DPPH method, intended for the determination of hydrophobic compounds only [28].

Moreover, in the literature, Sahiner showed different results of DPPH testing for rutin and poly(rutin) [27]. The polymeric form of rutin was characterized by a lower ability to reduce DPPH radicals than rutin. This difference was attributed to it having fewer OH functional groups compared to rutin. Active groups of poly(flavonoid) responsible for scavenging DPPH radicals could be connected by network nodes during polymerization, as a result of which their number decreased, and therefore the ability to scavenge DPPH radicals decreased.

The ability of naringenin and poly(naringenin) to reduce transition metal ions was assessed using the FRAP and CUPRAC methods. Naringenin before and after polymerization showed very little activity to reduce iron ions (FRAP method). In contrast to the FRAP assay, naringenin was characterized by a good ability to reduce ions of copper in the CUPRAC test. As a result of polymerization, the activity of naringenin to reduce Cu^2+^ ions decreased approximately five times. Mira and co-authors described similar results that naringenin did not have the ability to reduce Fe^3+^ ions, while it was characterized by good activity to reduce Cu^2+^ copper ions [39]. The activity of flavonoids to reduce and chelate metal ions is strictly dependent on their structure. The association between 5-OH and 4-oxo substituents in naringenin [9] contributes to the chelation of compounds such as heavy metals. There are no literature data on the structural elements of poly(flavonoids) responsible for the reduction ions of transition metal. In the case of the proposed cross-linking mechanism involving OH groups in the 5, 7 and 4′ position (Figure 2a), it seems reasonable to decrease the ability of poly(naringenin) to reduce Cu^2+^ ions. The poly(naringenin) OH groups in positions 5 and 4′ may take part in the crosslinking reaction and act as a link between the monomers; therefore, the activity measured by the CUPRAC method may be lower than the activity of the monomer.

In the literature, the polymeric catechin received as a result of the polymerization with the cross-linker GDE was characterized by a greater ability to reduce ions of iron (FRAP assay, about 2.7 times) and copper ions (CUPRAC test, around 3.9 times). Unlike poly(naringenin), the cross-linking reaction of poly(catechin) with GDE positively influenced the improvement of the compound’s ability to reduce ions of transition metal [28].

The antibacterial as well as antifungal activity of naringenin and poly(naringenin) are summarized in Table 3.

After 24 h of incubation, an increase in the number of all microorganisms was found in the control medium sample containing no flavonoid and poly(flavonoid). In the cultures with monomeric and polymeric naringenin, an increase in the number of *Escherichia coli* bacterial cells by about 1 to 1.8 logarithm was observed. This means that the samples tested did not have any antimicrobial activity against this microorganism. Naringenin and poly(naringenin) samples also showed no antibacterial activity against *Staphylococcus aureus* and *Bacillus subtilis*.

The increase in the number of *Candida albicans* yeast cells in flavonoid and poly(flavonoid) cultures was observed, but to a lesser extent than in the control sample. The dieback rate of the microorganisms D in the poly(naringenin) culture was 0.64 log values, indicating that the sample exhibited antimicrobial activity. Reference naringenin was characterized by a lack of such activity. The polymerization of this flavonoid had a very positive effect on its microbiological properties against *Candida albicans* yeast cells.

In addition, the activity of polyphenols against mold was investigated. The number of *Aspergillus niger* cells after 24 h increased by more than one row only in the control sample without flavonoid and poly(flavonoid). In the test tubes with naringenin and poly(naringenin), a decrease in the number of cells was noted in the cultures with both samples, indicating that they exhibited an antifungal effect. The dieback rate of microorganisms D in naringenin and poly(naringenin) cultures were −0.75 and −0.94 logarithm, respectively.

In summary, naringenin and poly(naringenin) had antimicrobial activity against mold *Aspergillus niger*, but only the polymeric form of naringenin had activity against yeast *Candida albicans*. The polymerization of naringenin with GDE resulted in a beneficial antimicrobial effect of naringenin against this microorganism.

In the scientific literature on the polymerization reactions of flavonoids with a cross-linking agent, Sahiner found that polymeric quercetin had stronger antibacterial activity than quercetin on *E. coli* ATCC 8739, *S. aureus* ATCC 25323 and *B. subtilis* ATCC 6633 strains [26].

The antimicrobial properties of catechin and its polymeric form received as a result of the reaction with GDE were also analysed [28]. The polymeric form of catechin showed a stronger antibacterial effect against *Staphylococcus aureus* than monomeric (+)-catechin, and antifungal activity against *Aspergillus niger* analogous to the activity of catechin.

As with the previous studies presented in this manuscript, the limited solubility of the compounds could also have an adverse effect on the results obtained in the antimicrobial tests. The lack of antimicrobial effect against *Escherichia coli*, *Staphylococcus aureus* and *Bacillus subtilis* may result from the low solubility of the samples in water, and therefore difficulty in penetrating the cells. Usually, polyphenols do not completely inhibit the growth of microorganisms; however, they prolong the adaptation phase, which was clearly visible in organisms that grow for longer, such as molds.

## 4. Conclusions

Poly(naringenin) was obtained by the polymerization reaction with GDE cross-linking agent. The strong cross-linking of the compound significantly hampered the analysis of the poly(flavonoid) structure because it was insoluble or only partially soluble in standard solvents used in studies such as NMR. The polymeric structure of the poly(naringenin) powder was confirmed by FTIR and UV-Vis techniques. The conversion of naringenin to poly(naringenin) was 85%, as shown by FTIR analysis. Poly(naringenin) had improved thermal properties compared to naringenin. The TG study showed that the final decomposition temperature, T60, of the polymeric naringenin was 94 °C higher than the T60 of naringenin. Moreover, the DSC test confirmed the higher resistance to oxidation of poly(naringenin). Poly(flavonoid) had a 13.8 °C higher final oxidation temperature, T_o,_ than monomeric naringenin. Determination of the free radical scavenging capacity of polyphenols showed that poly(naringenin) had a greater activity in reducing ABTS and DPPH radicals than the monomer. On the other hand, naringenin after polymerization was characterized by a lower ability to reduce copper ions. Poly(naringenin) showed antimicrobial activity against *Candida albicans* yeast, while the monomer had no activity against this microorganism. Both the monomer and the polymeric flavonoid had antimicrobial properties against *Aspergillus niger*.

Due to good oxidation resistance, high thermal stability as well as the ability to reduce free radicals and Cu^2+^ ions, poly(naringenin) can be proposed as a potential stabilizer, in, for example, environmentally friendly polymer materials. Moreover, thanks to its antimicrobial properties, the polymeric form of naringenin can potentially be used as a functional agent, for example, for polymeric packaging with antimicrobial properties.

## Figures and Tables

**Figure 7:**
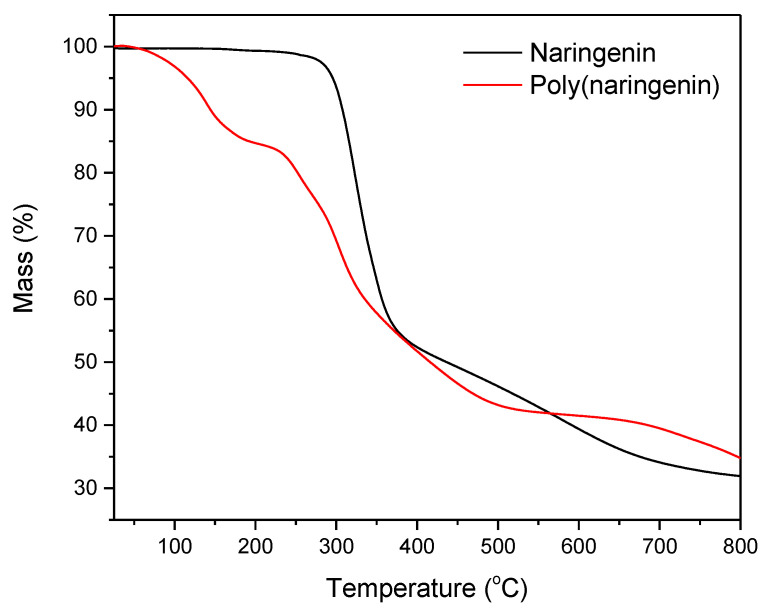
TG curves of naringenin and poly(naringenin).

**Table 1:** Values of T10, T50, and T60 of naringenin and poly(naringenin). Standard deviation: temperature ±0.7 °C.

Sample	T10	T50	T60
Naringenin	307	436	591
Poly(naringenin)	144	416	685

**Table 2:** DSC analysis of naringenin and poly(naringenin).

Sample	T_m_ (°C)	ΔH_m_ (J/g)	T_o_ (°C)	ΔH_o_ (J/g)
Naringenin	253.9	163.2	331.5 (endset)	87.5
Poly(naringenin)	45.1	425.1	345.3 (endset)	594.4

T_m_—melting point, ΔH_m_—melting enthalpy, T_o_—oxidation temperature, ΔH_o_—oxidation enthalpy, Standard deviations: temperature ±0.8 °C; enthalpy ±1.4 J/g.

**Table 3:** Antimicrobial properties of naringenin and poly(naringenin).

Sample	The Number of Microorganisms (cfu/cm^2^)		Log of the Number of Microorganisms		D
	t = 0	t = 24	t = 0	t = 24	
*Escherichia coli*					
Control medium	1.7 ± 0.21 × 10^6^	9.6 ± 1.56 × 10^7^	6.23 ± 0.05	7.98 ± 0.07	1.75
Naringenin		3.2 ± 0.36 × 10^7^		7.51 ± 0.05	1.27
Poly(naringenin)		8.0 ± 0.38 × 10^7^		7.90 ± 0.02	1.67
*Staphylococcus aureus*					
Control medium	6.5 ± 0.25 × 10^5^	1.1 ± 0.13 × 10^8^	5.81 ± 0.03	8.04 ± 0.10	2.23
Naringenin		9.6 ± 0.13 × 10^6^		6.98 ± 0.06	1.17
Poly(naringenin)		8.8 ± 0.81 × 10^7^		7.94 ± 0.04	2.13
*Bacillus subtilis*					
Control medium	1.3 ± 0.25 × 10^6^	1.6 ± 0.21 × 10^7^	6.11 ± 0.08	7.20 ± 0.06	1.09
Naringenin		1.0 ± 0.24 × 10^7^		7.00 ± 0.10	0.89
Poly(naringenin)		3.7 ± 0.93 × 10^7^		7.57 ± 0.11	1.45
*Candida albicans*					
Control medium	1.9 ± 0.38 × 10^5^	3.7 ± 1.47 × 10^6^	5.28 ± 0.08	6.57 ± 0.18	1.29
Naringenin		1.4 ± 0.48 × 10^6^		6.15 ± 0.16	0.87
Poly(naringenin)		8.2 ± 0.97 × 10^5^		5.91 ± 0.05	0.64
*Aspergillus niger*					
Control medium	1.3 ± 0.40 × 10^4^	2.4 ± 0.42 × 10^5^	4.11 ± 0.14	5.38 ± 0.08	1.27
Naringenin		2.3 ± 0.38 × 10^3^		3.36 ± 0.07	−0.75
Poly(naringenin)		1.5 ± 0.42 × 10^3^		3.18 ± 0.13	−0.94

## Data Availability

Data is contained within the article or Supplementary Material.

## Supplementary Materials

The following are available online at https://www.mdpi.com/article/10.3390/ma14092142/s1. Figure S1a: ^1^H NMR spectra of poly(naringenin) in deuterated water; Figure S1b: ^1^H NMR spectra of poly(naringenin) in DMSO.
