# Supplementary material for: Novel Polymeric Biomaterial Based on Naringenin
Source: Materials (Basel). 2021 Apr 23;14(9):2142. doi: 10.3390/ma14092142 (PMC8122925; doi:10.3390/ma14092142)
Supplement: Supplementary file 1 [file materials-14-02142-s001.zip › materials-1190186-supplementary.pdf]

materials

Supplementary Materials

Novel Polymeric Biomaterial Based on Naringenin

Malgorzata Latos-Brozio [1,*], Anna Masek [1] and Małgorzata Piotrowska [2]

1 Faculty of Chemistry, Institute of Polymer and Dye Technology, Lodz University of Technology, Stefanowskiego 12/16, 90-924 Lodz, Poland; anna.masek@p.lodz.pl
2 Faculty of Biotechnology and Food Sciences, Institute of Fermentation Technology and Microbiology, Lodz University of Technology, Wólczańska 71/173, 90-924 Lodz, Poland; malgorzata.piotrowska@p.lodz.pl
* Correspondence: malgorzata.latos@p.lodz.pl

Figure S1 a. ^1H NMR spectra of poly(naringenin) in deuterated water.

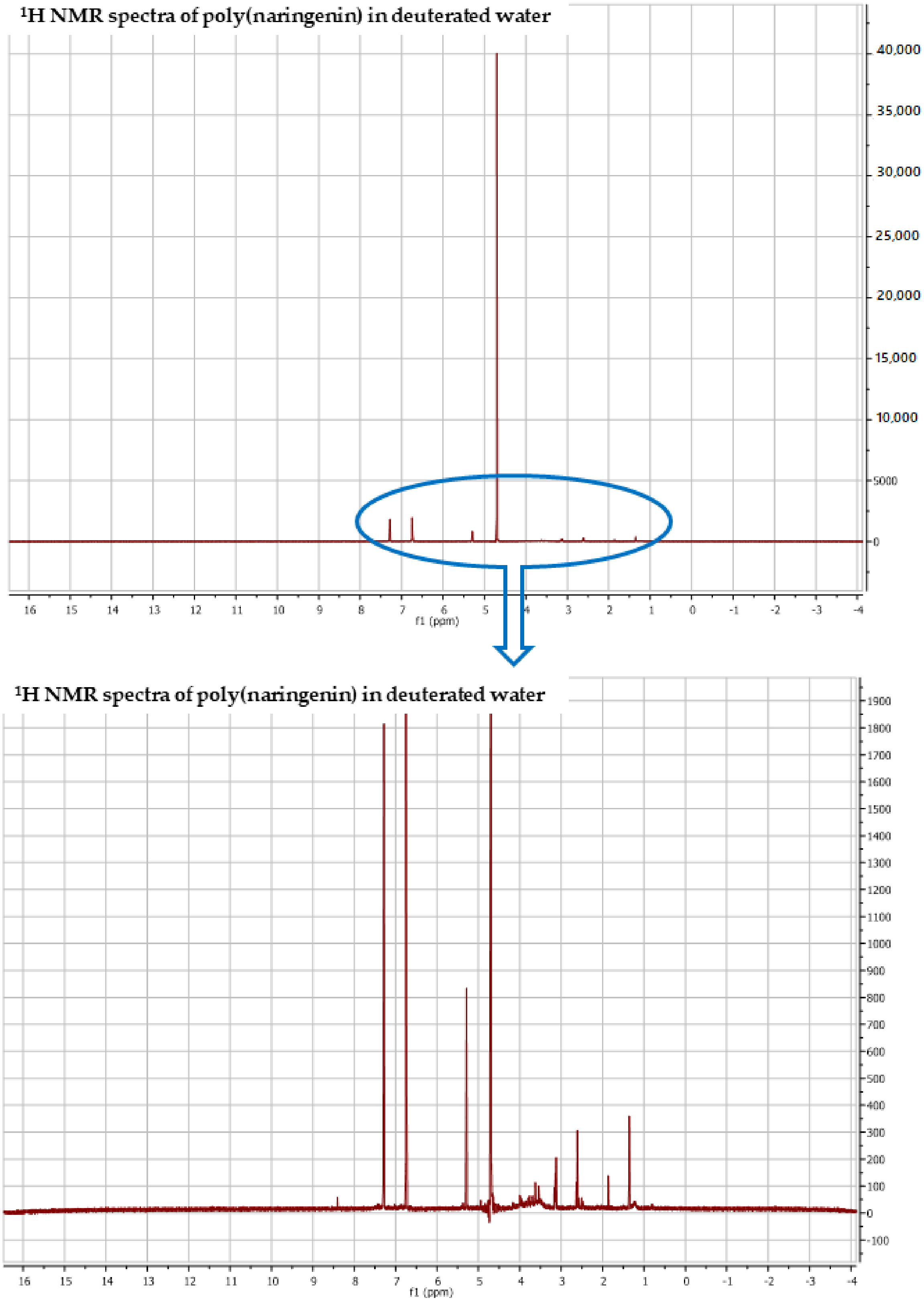

Figure S1a: ^1HNMR spectra of poly(naringenin) in deuterated water.

Figure S1 b. ^{1}H NMR spectra of poly(naringenin) in DMSO.

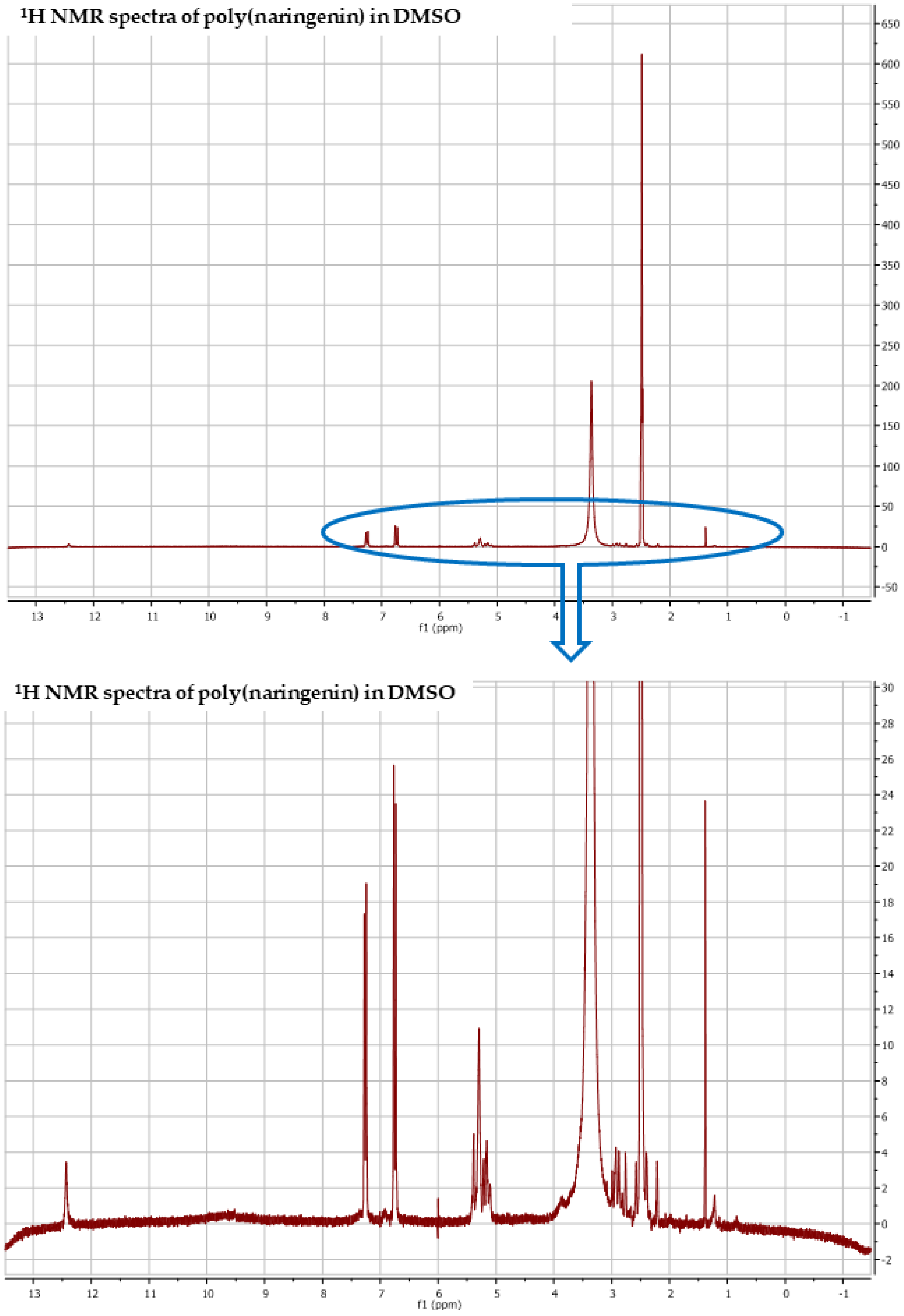

Figure S1b: ^{1}HNMR spectra of poly(naringenin) in DMSO.
